# The Influence of Fluoride Gels on the Physicochemical Properties of Tooth Tissues and Dental Materials—A Systematic Review

**DOI:** 10.3390/gels10020098

**Published:** 2024-01-26

**Authors:** Paweł J. Piszko, Aleksandra Piszko, Jan Kiryk, Adam Lubojański, Wojciech Dobrzyński, Rafal J. Wiglusz, Jacek Matys, Maciej Dobrzyński

**Affiliations:** 1 Department of Polymer Engineering and Technology, Faculty of Chemistry, Wroclaw University of Science and Technology (WUST), Wyb. Wyspiańskiego 27, 50-370 Wroclaw, Poland; 2 Department of Pediatric Dentistry and Preclinical Dentistry, Wroclaw Medical University, Krakowska 26, 50-425 Wroclaw, Poland; 3 Oral Surgery Department, Wroclaw Medical University, Krakowska 26, 50-425 Wroclaw, Poland; 4 Department of Dentofacial Orthopedics and Orthodontics, Division of Facial Abnormalities, Wroclaw Medical University, Krakowska 26, 50-425 Wroclaw, Poland; 5 Department of Organic Chemistry, Bioorganic Chemistry and Biotechnology, Faculty of Chemistry, Silesian University of Technology, Krzywoustego 4, 44-100 Gliwice, Poland; 6 Institute of Low Temperature and Structure Research, PAS, Okólna 2, 50-422 Wroclaw, Poland

**Keywords:** fluoride gels, dental materials, biomaterials, clinical applications, dentistry, teeth

## Abstract

The aim of the presented systematic review is to update the state of knowledge and relate the properties and composition of fluoride gels to their potential application. This article aims to explore the effect of fluoride gel application on changes in the properties of dental biomaterials and tooth tissues. The review includes articles assessing studies on the effects of fluoride gel on dental tissues and materials. Employing the PRISMA protocol, a meticulous search was conducted across the PubMed, Scopus, and Web of Science databases, utilizing keywords such as fluoride, gel, and properties. The publications were selected without limitation by the year of publication, and then Cohen’s κ test was used to assess the agreement of the respondents. Exclusion criteria included non-English studies, opinion pieces, editorial papers, letters to the editor, review articles and meta-analyses, clinical reports, studies lacking full-text accessibility, and duplicates. The quality of the chosen papers was assessed by two independent reviewers. A total of 2385 were located in databases, of which only 17 met the inclusion criteria. All publications showed increased surface mineralization, and seven studies showed the effect of fluoride gel on the surface of dental tissues. Three articles stated a negative effect of fluoride gels on titanium and stainless steel alloys and glass ionomer fillings. The effects on shear bond strength and plaque deposition require further investigation because the study results are contradictory.

## 1. Introduction

Modern dentistry is heading toward a minimally invasive and preventive approach. Among methods of prevention, the application of fluoride gels can be outlined. The impact of different preventive agents on dental tissues and other dental materials should be considered while selecting treatment methods. Fluoride foams, mouth rinses, and varnishes are alternatives to fluoride gels [1,2,3]. They differ in applicability and mode of action. However, in modern dentistry, emphasis is also placed on implementing techniques to diminish the consumption of dietary sugar, impede plaque metabolism, and enhance saliva functionality [4,5]. The presented systematic review puts emphasis on fluoride gels. Considering preventive therapy, the potential risks should also be taken into account. Therefore, a direct link between the composition of the dental material and its clinical efficacy shall be determined.

**Figure 1:**
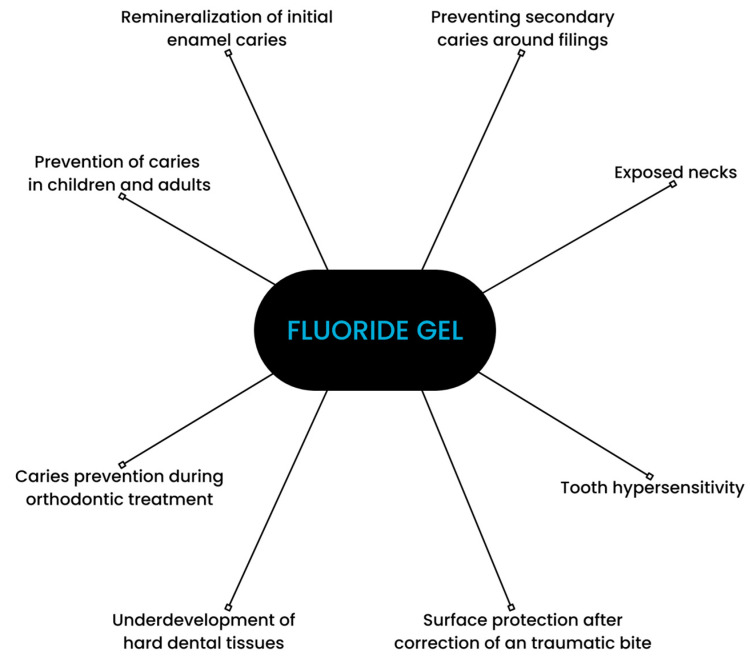
Scheme depicting applications of fluoride gel.

Fluoride gels are commonly recommended by professionals [6,7]. Their application is approved by the US Food and Drug Administration. Dental gels consist of fluoridated and acidic ingredients. Their pH usually ranges between about 3.2 and 7.7 [8,9]. Fluoride gels are available in the forms of acidulated phosphate fluoride (APF) and neutral sodium fluoride. The concentration of fluoride ranges from 1000 to 12,300 ppm. They can be applied at a dental office by a medical professional or self-applied [10]. Fluoride gels are recommended in cases of enamel demineralization, dentine hypersensitivity, and moderate or high caries risk, e.g., among patients with xerostomia, fixed orthodontic appliances, and vulnerable elders [11,12]. A study comparing fluoride gel to a placebo exhibited a preventive fraction of 21% [13]. The main areas of fluoride gels’ application are depicted in Figure 1.

Fluoride plays a vital role in caries prevention. However, its mode of action is not fully understood. It is an important factor in the processes of demineralization and remineralization [6,14,15]. When present in saliva in a sufficient amount, fluoride ions delay demineralization and promote enamel remineralization [16,17]. Fluoride present in the solution surrounding the enamel crystals is able to adsorb to the surface of the carbonated apatite crystals, inhibiting demineralization. The mechanism of enamel remineralization consists of replacing the –OH groups of hydroxyapatite with fluorine [18]. During this process, fluorine ions become incorporated into the apatite structure, forming fluor–hydroxyapatite mixed crystals, which are more resistant to future acid challenges [19]. Fluoride may also affect bacteria’s metabolism and adherence to the enamel. Fluoride ions and hydrofluoric acid can bind directly to many enzymes, for example, heme-containing enzymes or other metalloenzymes, to modulate metabolism [20].

The incorporation of fluoride into dental biomaterials facilitates its controlled release into hard tissues and the environment of the oral cavity. Antimicrobial and cariostatic properties of dental biomaterials are frequently associated with the amount of released fluoride [14,21,22]. Moreover, in order to be applicable in the oral cavity, biomaterials have to be biocompatible, as broadly defined by David F. Williams [23]. Physicochemical properties are also vital in the case of dental biomaterials. For example, wettability affects cellular adhesion and interaction with liquids [24]. Although fluoride gels help decrease the occurrence of caries in orthodontic patients, recent research indicates that their application may adversely affect the mechanical properties of orthodontic wires, highlighting the need for a balanced approach to treatment planning [25]. Furthermore, fluoride gels may affect the structure of restorations (e.g., glass ionomers) depending on the pH and form of fluoride incorporation (APF, neutral sodium fluoride) [26].

The literature reports multiple studies on the impact of various dental materials on dental tissues [27,28,29]. However, it lacks up-to-date systematic reviews on the impact of fluoride gels, and available review articles regarding fluoride gels are older than 8 years [11,13,30,31]. In recent advancements, researchers have discovered novel formulations that show promising results in enhancing the effectiveness of fluoride gels. These emerging formulations are crucial to consider in understanding the comprehensive landscape of fluoride gel applications. Therefore, the aim of the presented article is to update the state of knowledge and relate the properties and composition of fluoride gels to their potential application. This systematic review summarizes and concludes the impact of fluoride gels on the physicochemical properties of tooth tissues and dental materials.

## 2. Materials and Methods

### 2.1. PRISMA Statement

We completed the PRISMA 2020 checklist and constructed a flowchart, following the PRISMA guidelines. The selection process was based on the PRISMA 2020 statement [32].

### 2.2. Focused Question

The systematic review followed the PICO framework as follows:

PICO question: In the case of tooth tissues and dental materials (population), will the application of fluoride gel (investigated condition) cause a change in their properties (outcome) compared to tooth tissues and dental materials without fluoride gel application (comparison condition)?

### 2.3. Protocol

**Figure 2:**
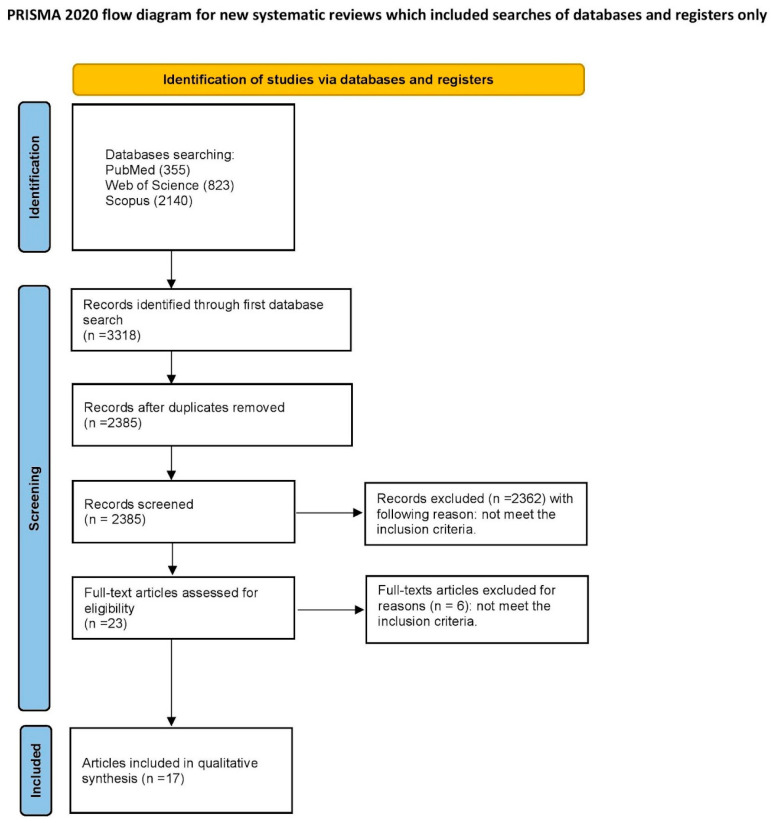
PRISMA 2020 flow diagram of the study.

The article selection process for the systematic review was outlined carefully, following the PRISMA flow diagram [32]—presented in Figure 2.

### 2.4. Eligibility Criteria

The review contains articles examining studies investigating the impact of fluoride gel on tooth tissues and dental materials, both in vitro and in vivo studies, studies published in English, and studies with a control group. After deliberating as a group, the reviewers eliminated research that satisfied the subsequent standards: non-English studies, opinion pieces, editorial papers, letters to the editor, review articles and meta-analyses, clinical reports, studies lacking full-text accessibility, and publications that were duplicated. There were no limitations placed on the year of release.

### 2.5. Information Sources, Search Strategy, and Study Selection

On 6 November 2023, a systematic electronic search was performed across multiple scholarly databases, including PubMed, Web of Science (WoS), and Scopus. In PubMed and WoS, our focus was specifically on titles, authors, and abstracts, while in the Scopus database, we meticulously narrowed down our search to titles, authors, and keywords. The search criteria were meticulously crafted, centering around the precise keywords ((fluoride) AND (gel) AND (properties)). Following the database search, a literature search was conducted to find any papers that were deemed unfound for the study. Only articles with full-text versions were taken into consideration.

### 2.6. Data Collection Process and Data Items

Two reviewers autonomously gathered data from articles that fulfilled the inclusion criteria. The extracted information was then entered into a standardized Excel file.

### 2.7. Assessing Risk of Bias in Individual Studies

At the first stage of selecting studies, the titles and abstracts of each study were independently examined by the authors to minimize bias. Cohen’s κ test detects agreement between reviewers [33]. Distinctions among authors’ conceptions of inclusion or exclusion of articles were discussed between reviewers. The risk of bias was assessed based on the scoring assigned during the Quality Assessment.

### 2.8. Quality Assessment

Two independent reviewers (P.J.P. and J.M.) rated the standard of each study’s quality in the article. The evaluation standards were predicated on the existence of crucial data related to the influence of fluoride gel on tooth tissues and dental materials. To ensure the value of the study design, implementation, and analysis, the following criteria were used: a minimum group size of 10 subjects, the presence of a control group, sample size calculation, the composition of gel, consideration of the type of examined surface, and analysis of the effect of fluoride gel on tooth tissues or dental material surfaces, including parameters such as remineralization and loss of material hardness and presentation of clinical applicability. The studies were scored on a scale of 0 to 6 points, with a higher score indicating higher study quality. The risk of bias was assessed as follows: 0–2 points denoted a high risk, 3–4 points denoted a moderate risk, and 5–6 points indicated a low risk. Any discrepancies in scoring were resolved through discussion until a consensus was reached.

## 3. Results

### 3.1. Study Selection

An initial search of the database identified 2385 articles that were potentially eligible for the literature review. After screening the titles and abstracts, 2362 articles were excluded as they were unrelated to the reviewed topic. Among the remaining 23 articles, there were no duplicates. After a full-text examination, six articles were rejected due to not meeting the inclusion criteria. Finally, 17 articles qualified for the systematic review [8,12,24,25,34,35,36,37,38,39,40,41,42,43,44,45,46]. All of the included studies were written in English and were either in vitro or in vivo studies. Their scope was the influence of fluoride gels on the physiochemical properties of dental materials and tissues.

### 3.2. General Characteristics of the Included Studies

Seventeen articles were included in this review. The general characteristics of each study (including fluoride gel composition utilized material/evaluated tissue and conclusion) are presented in Table 1.

In orthodontic treatment with fixed appliances, the mechanical properties of archwires are crucial for proper therapy. Taqa et al. [34] used stainless steel and nickel–titanium archwires in their study, which examined the influence of acidulated phosphate with 1.23% fluoride ions and a pH of 3.5. Gupta et al. [25] examined the effect of 1.1% sodium acidulated phosphate fluoride, APF, which had 0.5% *w*/*v* fluoride and a pH of 5.1. Both articles demonstrate changes on the surface of the arches through SEM images, which may compromise their strength.

One study [38] investigated the shear bond strength between composite resin and various metal alloys. Acidulated phosphate fluoride gel with a fluorine content of 1.23% and a pH of 3.0 was used, but no statistically significant changes in bond strength were noticed. Toumelin-Chemla et al. [8] conducted research using a gel containing 0.553 g of NaF and 1.126 g of NH_4_F, pH 5.5, on the surface of the titanium alloy. As in previous studies, the titanium alloy degraded significantly. In a review of articles examining the effects of fluoride gel on metals and alloys, one in vivo study investigated a group of fifteen dental students with orthodontic brackets made of titanium alloy and stainless steel. In the study, fluoride gel containing 0.4% stannous fluoride with a pH of 3.2 was used. The authors reported no significant changes in the structure of the arches, which can be attributed to the presence of saliva and fluids in the oral cavity [44]. It is important to note the formation of a coating by saliva, which can significantly affect the effectiveness of fluorine compounds on the surface of the tested material [47].

Two of the mentioned works investigated the effects of fluoride gels on bovine tissues. Gruba et al. [35] used hydrogen peroxide (H_2_O_2_) with 0.1% F, H_2_O_2_ + 1% sodium trimetaphosphate nano (TMPnano), and H_2_O_2_ + 0.1% F + 1% TMPnano in their research. The presence of fluoride resulted in fewer surface structure changes in bovine enamel and dentin discs compared to the control group. Other studies carried out in similar conditions (with the concentration of fluorine increased twofold) demonstrated that the higher the fluoride concentration during bleaching, the less negative impact it has on the mechanical properties of the surface [48]. Another study utilized colorless 2% NaF gel, blue 2% NaF gel, and pink 2% NaF gel. The aim of the study was to determine whether the pigment in the gel affects tissue color. According to the authors’ research, there were no changes in tissue color [37].

Five studies were conducted on human teeth, with Srinivas et al. examining the effect of topical acidulated phosphate fluoride (APF) gel on the mean contact angle [24]. The results showed less wettability in the anterior teeth. Yasar et al. investigated the effect of fluoride gel (1.23% APF), mineralization-promoting peptide-3 gel (10% MPP3), and a combination of fluoride gel+MPP3 and 1.23% APF + 10% MPP3 on primary molars. The combination of APF and MPP3 provided the most effective protection against caries and demonstrated remineralizing properties [39]. Two studies investigated the effects of adding 0.463% sodium fluoride to 10% carbamide peroxide whitening gel on the lower third molars. Both studies found that the addition of a fluorine compound did not affect the whitening properties but did increase tissue remineralization [40,42]. Reddy et al. demonstrated remineralization of enamel on extracted premolars after the application of 1.23% APF gel [43].

In their study, Wiglusz et al. created novel gels containing fluoride and studied fluoride release at different pH levels. The results showed that lower pH levels resulted in higher fluoride ion release [12]. In Wade et al.’s article, 0.4% stannous fluoride gel was used and exhibited the lowest level of bacterial growth inhibition compared to other tested agents [36].

Glass ionomer cements are commonly used in conservative dental treatment due to their ability to store fluoride. Gill et al. found a statistically significant reduction in material strength when using 1.23% APF gel and 2% NaF gel [41]. Kula et al. investigated the effect of 1.23% APF gel, 0.5% APF gel, 0.4% SnF2 gel, and 1.1% NaF gel on resin composite. The highest loss was observed in gels containing APF, and the lowest in the case of NaF [45]. Addy et al. conducted an in vivo study on fifteen participants who used several agents, including commercial stannous fluoride gel (0.4%), for four days. The chlorhexidine rinse was the most effective in preventing plaque growth, with minimal differences observed between other measures [46].

### 3.3. Main Study Outcomes

Seven studies [24,35,37,39,40,42,43] demonstrated the effect of fluoride gel on the tissue surface of human or bovine teeth, all of which showed increased surface remineralization. Three articles [8,25,34] also demonstrated the effect of fluoride gel on the surface of stainless steel and titanium alloys, with defects observed in all cases. Additionally, one in vivo study [44] was conducted on the surface of stainless steel and metal alloys. No statistically significant changes were found on the surface in this case. Lim et al. [38] found no significant effect of fluoride gel on shear bond strength. However, another study [49] examining the effect of various fluoride gels on resin composite showed material deterioration. A research study [41] found a negative influence of fluoride gels on glass ionomer cements. A study [12] developed new fluoride gels and demonstrated an increased release of fluoride ions at lower pH. An article [36] on the effects of various fluoride-containing substances revealed that fluoride gels have the narrowest range of action against bacteria. Another in vivo study [46] found that the impact of fluoride gels on dental plaque deposition was not significantly different from that of other agents tested, except for mouthwash containing chlorhexidine, which was by far the most effective.

### 3.4. Quality Assessment

Out of seventeen articles, ten were assigned with a low risk of bias [34,35,37,38,39,40,42,43,45,46], six with a moderate risk of bias [8,12,25,36,41,44], and one with a high risk of bias [24]. The results of the performed Quality Assessment are presented in Table 2.

## 4. Discussion

Fluoride-containing products are fundamental in dental prevention. The impact of fluoride gel on the surface of metal alloys included in this study was found to be negative.

When considering compounds containing fluorine, it is important to take into account the pH level as it greatly affects the formation of crevices.

Metals used in dentistry, such as titanium and alloys, can be damaged by agents with a pH below 3.5. These metals are widely used in prosthetics, orthodontics, and dental surgery. Therefore, it is important to provide dental products with the correct pH [50,51]. Regarding nickel content in metal alloys, the tests showed a significant difference in the surface structure in favor of nickel-free alloys in comparison with stainless steel after the application of fluorine-containing agents [52,53].

The concentration of fluorine ions in dentistry agents is directly proportional to the level of damage caused to the surface of metals and their alloys due to the decrease in pH [54]. In the case of composite materials, the type of gel used is a crucial factor. Despite pH, Yeh et al. found statistically significant differences in the roughness and microhardness of the composite surfaces among the three types of fluoride gels used. The authors suggested that the presence of magnesium aluminum silicate in two of the gels may have prevented damage to the surface [55]. The effectiveness of fluoride in gels in preventing caries is well established. This prevention is especially crucial for children and adolescents who often struggle with proper hygiene [13].

As mentioned, while comparing the impact of fluoride gels on the properties of dental tissues or materials, the concentration of fluoride and gel composition should be considered. It is worth noting that some of the articles included in this systematic review lack complete information regarding the composition of gels used in the study. However, the available information is sufficient to draw meaningful conclusions.

However, the vast majority of them mention the specifications of the fluoride products used. A few studies determined only the fluoride concentrations in the gels, among which the most popular is 1.23% [34,38,39,43]; also, a concentration of 0.4% may be listed [36,46]. Kula et al. compared the impact of four different fluoride gels with various compositions detailed in the work [45]. The analyzed formulations were based on 1.1% sodium fluoride (0.5% F, *w*/*v*), 2.6% sodium fluoride, 0.17% hydrofluoric acid (1,23% F, *w*/*v*), and 0.4% stannous fluoride (0.09%F, *w*/*w*). After comparison of the impact of various gels on the degradation of composite materials, the authors noticed significant differences and concluded that topical application of APF agents resulted in a substantial reduction in filler content from the resin composite specimens, while 1.1% NaF produced the least amount of damage [45].

Some studies have examined bleaching gels that contain fluoride. These gels are expected to have various therapeutic effects, but their impact on dental tissues and materials may differ [35,40,42]. When using whitening gels, the type and concentration of the bleaching agent, as well as the fluoride concentration, may have an impact on dental tissues. The evaluated studies used peroxides as the bleaching agents. The products were based on either 10% carbamide peroxide [42] or 35% hydrogen peroxide with the addition of sodium fluoride and nano-sized sodium trimetaphosphate [35]. Gladwell et al. compared bleaching using gel with and without fluoride. The authors concluded that the addition of fluoride to a tooth-whitening system has no impact on the gel’s whitening effectiveness. However, the inclusion of fluoride may offer remineralization benefits to the gel [42].

There are numerous fluoride-containing agents used in dentistry, differing in their fluorine content and method of application. Fluoride varnishes have a high concentration of fluoride content and may stay on the tooth surface for a long time. In the case of in vitro tests, the maintenance of hygiene and slowing down the release of fluoride through saliva were not taken into account [56].

Fluoride prevention is essential in dentistry, in addition to proper oral hygiene. The ability of fluoride to remineralize and increase tissue resistance to lower pH, as well as its antibacterial properties, determines its importance. This is particularly crucial in cases where maintaining proper hygiene is difficult, such as with fixed braces, prosthetic restorations, or implants. It is strongly recommended to follow fluoride prophylaxis in these cases [57,58].

The environment of the oral cavity is influenced by a range of chemical, physical, and biological factors. Therefore, it is important to note that a significant limitation of the presented study is the inability to directly extrapolate in vitro findings into in vivo settings and vice versa. Another limitation of this study is the lack of complete information on the manufacturers and detailed compositions of the fluoride gels used in the referenced studies.

## 5. Conclusions

The presented results answer the posed PICO question: In the case of tooth tissues and dental materials, the application of fluoride gels causes a change in their properties in comparison to reference material without a fluoride agent.

Gels containing acidulated phosphate fluoride ions may reduce the mechanical properties of archwires made of NiTi alloys and stainless steel. However, the surface of metal orthodontic arches is protected against the action of gel containing tin fluoride by saliva and the environment of the oral cavity, as confirmed in clinical conditions.

The application of gels with the addition of APF also reduces the mechanical strength of glass ionomers and composite materials used in restorative dentistry. According to in vitro studies, an increase in fluoride concentration in bleaching gels reduces enamel demineralization. Furthermore, the caries-protective and mineralization-promoting effects of fluoride were enhanced by the addition of MPP3 peptide to fluoride gel.

In light of these findings, dentists should be aware of the diverse effects of fluoride gel composition on various dental materials. Understanding these implications is crucial for dentists to make informed decisions and effectively incorporate fluoride gels into their clinical practice for optimal patient care.

## Figures and Tables

**Table 1:** General characteristics of the selected studies.

Reference	Fluoride Gel Composition	Dental Material	Dental Tissue	Type of Study	Conclusions and Clinical Applicability
Taqa et al. [34]	Acidulated phosphate fluoride (APF) gel and TOPEX acidulated phosphate with 1.23% fluoride ions and pH of 3.5 (Sultan Healthcare Inc., York, PA, USA)	40 stainless steel samples (SS remanium) and nickel–titanium orthodontic wires	n/a	in vitro	The fluoride gel had no effect on the tensile properties of the wires. SEM revealed significant degradation of the surface of both SS remanium and NiTi wires after immersion in fluoride gel.
Srinivas et al. [24]	Topical acidulated phosphate fluoride (APF) gel	n/a	20 teeth (10 anterior and 10 posterior)	in vitro	The average contact angle of the topical fluoride APF gel on the anterior tooth surface is higher compared to the posterior tooth surface. This indicates increased wettability on the posterior tooth surfaces.
Wiglusz et al. [12]	3 gels (G-F, G-F-nFAP, and G-nFAP gel): G-F gel contained 4% *w*/*w* NaF (with F-20,000 ppm), G-F-nGAP gel consisted of 4% *w*/*w* NaF and 10% w/w nFAP, G-nFAP gel only had 10% *w*/*w* nFAP	n/a	n/a	in vitro	Viscosity of the formulations impacts the mechanism of fluoride release. The fastest release of fluoride ions was observed in the gel G-F specimen. The solubility of the substance contained in the hydrogel and its subsequent drug release is determined by the amount of free water and water bound to the hydrogel. Addition of nHAp and NaF shows potential to reduce dental hypersensitivity through preventative and managed approaches.
Gruba et al. [35]	35% hydrogen peroxide (HP); H_2_O_2_ + 0.1% F (HP/F); H_2_O_2_ + 1% TMP nano (HP/TMPnano); H_2_O_2_ + 0.1% F + 1% TMPnano (HP/F/TMPnano); H_2_O_2_ + 2% calcium gluconate (HP/Ca)	n/a	Bovine enamel/dentin discs (n = 180)	in vitro	Gels containing F/TMPnano do not interfere with the whitening effect. They also reduce enamel demineralization, H_2_O_2_ diffusion, roughness, and morphological alterations. Whitening gels containing F/TMPnano can be used to improve safety and enhance clinical performance.
Wade et al. [36]	Gel with 0.4% stannous fluoride (Colgate Palmolive, New York, NY, USA)	n/a	n/a	in vitro	All evaluated samples exhibited antibacterial activity. The difference in color suggested that the availability of stannous ions in the formulations varied.
Vieira et al. [37]	3 experimental groups according to the color of fluoride gel (Fluorsul gel, Iodontosul; Porto Alegre, RS, Brazil): colorless 2% sodium fluoride (NaF) gel; blue 2% NaF gel; pink 2% NaF gel)	n/a	10 freshly extracted bovine incisors	in vitro	Discoloration of enamel does not occur during treatment with fluoride gel—independent of the presence of colored pigments in the product. Using colored fluoride gels poses no risk of enamel staining.
Lim et al. [38]	Commercially available acidulated phosphate fluoride gel (60 Second Topical APF Gel, Pascal Co., Arlington Heights, IL, USA) of 1.23% fluoride concentration (pH 3.5)	CPTI CP-titanium; TI64 Ti-6Al-4V alloy; TZSN experimental alloy (Ti-6Zr-6Sn-6Nb); TZN experimental alloy (Ti-13Zr-6Nb); NiTi experimental alloy (Ni-Ti); CTL Vitallium (Co-Cr-Mo)—surface covered with light-cured composite resin (Z100, 3M, USA)	n/a	in vitro	Bond strengths similar to those of sandblasted titanium alloys have been achieved by surface treatment with acid fluoride gel. The bond strength between titanium alloys and composite resins was not affected by aging in the low-concentration fluoride solution. Fluoride gel treatment can be used as an alternative to sandblasting to improve the bond strength of composite resin to titanium alloys.
Yaşar et al. [39]	Fluoride gel (1.23% APF); MPP3 gel (10% MPP3); fluoride gel + MPP3 (1.23% APF + 10% MPP3)	n/a	60 decay-free primary molars collected from children aged 6–12 years	in vitro	The combination of MPP3 and fluoride gel has increased the protective effect against caries as well as mineralization.
Bollineni et al. [40]	10% carbamide peroxide gel (VivaStyle, Ivoclar Vivadent AC); 0.463% sodium fluoride added to 10% carbamide peroxide whitening gel (VivaStyle, Ivoclar Vivadent AC)	n/a	24 lower third molar teeth, sectioned into quadrants	in vitro	The addition of 0.46% fluoride to 10% carbamide peroxide gel induced enamel remineralization in comparison with unfluoridated 10% carbamide peroxide gel. Addition of fluoride to a 10% carbamide peroxide gel did not show any significant impact on the gel’s whitening efficacy. Whitening properties are comparable for both fluoridated and non-fluoridated gels.
Gill et al. [41]	Topex—1.23% APF gel (Sultan Dental Products, Englewood, NJ, USA); 2% NaF gel pH 7 Neutral Gel (Pascal International Inc., Bellevue, WA, USA)	Glass ionomer cements: Fuji II (GC Corporation, Tokyo, Japan); Ketac Fil Plus (3M ESPE, Seefeld, Germany) High-viscosity glass ionomer cements: Ketac Molar Easymix (3M ESPE, Seefeld, Germany); Fuji IX GP (GC Corporation, Tokyo, Japan) Resin-modified glass ionomer cements: Vitremer (3M ESPE, St. Paul, MN, USA); Fuji II LC (GC Corporation, Tokyo, Japan)	n/a	in vitro	APF gel with a concentration of 1.23% caused a statistically significant reduction in microhardness in comparison with the control group. Decrease in microhardness was more pronounced in conventional glass ionomer cements and less pronounced in resin-modified glass ionomer cements. No significant difference in microhardness was observed after NaF. Application of 1.23% APF gel has the potential to reduce longevity of glass ionomer restorations.
Gladwell et al. [42]	10% carbamide peroxide whitening gels: fluoride free (group A) and with fluoride (0.463%NaF, group B)	n/a	24 extracted teeth (third molar), sectioned into quadrants	in vitro	The inclusion of fluoride into a tooth bleaching regimen has no effect on the gel’s ability to whiten teeth. Addition of fluoride to the gel enhances remineralizing properties.
Reddy et al. [43]	APF gel (Patterson NE. International, St. Paul, MN, USA)	n/a	100 extracted premolars, free of caries and enamel cracks	in vitro	All examined materials (including the APF gel) reduced progressive demineralization in comparison with the control group.
Gupta et al. [25]	Phos-Flur gel (1.1% sodium acidulated phosphate fluoride, APF, 0.5% *w*/*v* fluoride, pH = 5.1; Colgate Oral Pharmaceuticals)	The commercially available round preformed NiTi orthodontic archwire (3M, Inc., St. Paul, MN, USA)	n/a	in vitro	Irrespective of the composition of the wire, fluoride mouth rinses and gels affect the structural surface quality and strength of wires used in orthodontic treatment.
Harzer et al. [44]	Gel Kam toothpaste (Colgate, Hamburg, Germany) containing soluble stannous fluoride (pH 3.2)	Patients received a fixed orthodontic appliance consisting of bands attached to the molars and brackets attached to the incisors, canines, and premolars. Titanium brackets and stainless steel brackets were used for the study	n/a	in vivo	The simultaneous use of titanium brackets, acidic fluoride toothpastes, and fluoridated foods is completely safe for titanium brackets. It will not cause corrosion.
Toumelin-Chemla et al. [8]	Fluoridated odontological gel (Fluogel, Labo, Dentoria, Orleans, France) of which the composition is NaF; NH_4_F; potassium sorbate	n/a	n/a	in vitro	Titanium is a metal that has excellent corrosion resistance. However, it suffers significant degradation in fluoridated acidic media.
Kula et al. [45]	1.23% APF gel; 0.5% APF gel; 0.4% SnF2 gel; 1.1% NaF gel	Fifty specimens of a commercial barium boroalumino- silicate glass and silica-filled composite (Fulfil, Caulk Co., Milford, DE, USA)	n/a	in vitro	Topical APF agents resulted in significant filler loss from the resin composite specimens, while 1.1% NaF caused the least amount of deterioration.
Addy et al. [46]	Commercial stannous fluoride gel (0.4%)	A group of 15 male (6) and female (9) individuals aged between 20 and 27 years were selected as volunteers	n/a	in vivo	Compared with the toothpaste and gel products, plaque regrowth was significantly reduced with the CHX rinse and significantly increased with the saline rinse.

**Table 2:** Quality Assessment.

Reference	Group Size of Min. 10 Samples	Sample Size Calculation	Control Group	Description of Gel Composition	Description of the Effect of Fluoride Gel on Tooth Tissues or Dental Material Surfaces	Description of Potential Clinical Applicability	Total Points	Risk of Bias
Taqa et al. [34]	0	1	1	1	1	1	5	Low
Srinivas et al. [24]	1	0	0	0	1	0	2	High
Wiglusz et al. [12]	0	0	1	1	1	0	3	Moderate
Gruba et al. [35]	1	0	1	1	1	1	5	Low
Wade et al. [36]	0	0	1	1	1	1	4	Moderate
Vieira et al. [37]	1	0	1	1	1	1	5	Low
Lim et al. [38]	1	0	1	1	1	1	5	Low
Yaşar et al. [39]	1	1	1	1	1	1	6	Low
Bollineni et al. [40]	1	0	1	1	1	1	5	Low
Gill et al. [41]	0	0	1	1	1	0	3	Moderate
Gladwell et al. [42]	1	0	1	1	1	1	5	Low
Reddy et al. [43]	1	0	1	1	1	1	5	Low
Gupta et al. [25]	0	0	1	1	1	1	4	Moderate
Harzer et al. [44]	0	0	1	1	1	1	4	Moderate
Toumelin-Chemla et al. [8]	0	0	1	1	1	1	4	Moderate
Kula et al. [45]	1	0	1	1	1	1	5	Low
Addy et al. [46]	1	0	1	1	1	1	5	Low

## Data Availability

The data presented in this study are openly available in scientific databases under enlisted reference positions.
